# Coagulation Factor IIIa (*f3a*) Knockdown in Zebrafish Leads to Defective Angiogenesis and Mild Bleeding Phenotype

**DOI:** 10.3389/fcell.2022.852989

**Published:** 2022-03-21

**Authors:** Saravanan Subramaniam, Jiandong Liu, Craig Fletcher, Ramani Ramchandran, Hartmut Weiler

**Affiliations:** ^1^ Department of Medicine, Pulmonary Center, Boston University School of Medicine, Boston, MA, United States; ^2^ Blood Research Institute, Blood Center of Wisconsin: Part of Versiti, Milwaukee, WI, United States; ^3^ UNC McAllister Heart Institute, University of North Carolina at Chapel Hill, Chapel Hill, NC, United States; ^4^ Department of Pathology and Laboratory Medicine, University of North Carolina at Chapel Hill, Chapel Hill, NC, United States; ^5^ Department of Pediatrics, Division of Neonatology, Medical College of Wisconsin, Milwaukee, WI, United States

**Keywords:** tissue factor, bleeding, angiogenesis, *f3a*, morpholinos

## Abstract

Tissue factor (TF) is crucial for embryogenesis, as mice lacking TF are embryonically lethal (E10.5). This lethality may be attributed to defects in vascular development and circulatory failure, suggesting additional roles for TF in embryonic development beyond coagulation. In this study, we characterized the role of one of the TF paralogs (*f3a*) using a zebrafish model. The expression of *f3a* during embryonic developmental stages was determined by RT-PCR. Spatiotemporal expression pattern of *f3a* revealed (high expression from 28 to 36 hpf) the role of in the development of the yolk sac, circulation, and fins. Morpholinos (MO), an antisense-based oligonucleotide strategy, was used to knockdown *f3a* and examined for defects in morphological appearance, bleeding, and vascular patterning. *f3a* MO-injected embryos showed morphological abnormalities, including shorter body lengths and crooked tails. O-dianisidine staining showed *f3a* MO-injected embryos exhibited bleeding in the trunk (5.44%) and head (9.52%) regions. Imaging of endothelial-specific transgenic lines (*flk1:egfp-NLS/kdrl:mCherry-CAAX*) showed a 3-fold decreased caudal vein plexus (CVP) in *f3a* morphants versus controls at 48 hpf, suggesting a potential role for *f3a* in angiogenesis. These findings confirm that *f3a* is essential for angiogenesis, in addition to its involvement in hemostasis.

## Introduction

Tissue factor (coagulation factor III), a cell surface transmembrane glycoprotein receptor for coagulation factor VII/FVIIa, is a well-recognized trigger for the extrinsic coagulation cascade. The human tissue factor (TF) gene was cloned in late 1980s (Morrissey et al., 1987; Spicer et al., 1987; Mackman et al., 1989) and is localized on chromosome 1, p21-p22. TF is involved in inflammation, thrombosis, atherosclerosis, sepsis, tumor progression, embryogenesis (Pawlinski and Mackman, 2004; Chen and Dorling, 2009), and maintenance of vascular integrity in the placenta (Erlich et al., 1999). Disruption of the TF gene in mice is associated with embryonic lethality by E10.5 (Bugge et al., 1996; Carmeliet et al., 1996; Toomey et al., 1996), consistent with the suggestion that humans cannot survive without TF (Tuddenham et al., 1995). Because TF-deficient mice are embryonically lethal, researchers who investigated embryonic developmental stages up to E10.5 have hypothesized that the lethality might be due to a defect in vascular development (Carmeliet et al., 1996) and circulatory failure (Bugge et al., 1996; Toomey et al., 1996). Therefore, although TF is essential for embryonic development, its specific functions are not well defined due to embryonic lethality.

Angiogenesis is the growth of blood vessels from the existing vasculature and occurs throughout life, in health and disease conditions. The role of TF in tumor angiogenesis has been investigated for over 2 decades. One study concluded that the cytoplasmic domain of TF regulates the production of vascular endothelial growth factor (VEGF) (Chen et al., 2001; Belting et al., 2004; Zhang et al., 2005). Another study involving TF-deletion in mice concluded that yolk sac vessels of TF-deficient embryos were more fragile due to a deficit in mesenchymal cells/pericyte accumulation (Carmeliet et al., 1996). Embryonic lethality in mice has made investigations into the physiological role of TF in vascular development a bit challenging. Complementary to mouse model, the vertebrate zebrafish model system is used to investigate vascular development (Tonelli et al., 2020). Zebrafish embryogenesis studies have played a significant role in understanding the development of the embryonic vasculature. Interestingly, zebrafish can survive the first week of development without a functional vasculature or heartbeat, unlike mammalian embryos which cannot survive without a functional cardiovascular system (Stainier et al., 1995; Stainier, 2001; Sehnert et al., 2002; Jin et al., 2007). This viability allows us to perform detailed studies even in zebrafish with severe cardiovascular defects. In the zebrafish genome, approximately, 20% of the genes are duplicated. For TF, zebrafish has 2 TF paralogs, designated as *f3a* and *f3b* (Howe et al., 2013), which are homologous to human and mouse TF genes (Stein et al., 2007), respectively. Knockdown of *f3b* using a morpholino oligonucleotide (MO) suggests that the *f3b* gene is important for coagulation and angiogenesis (Zhou et al., 2011). However, there are no reports on the role of *f3a* till date. In this study, we were able to successfully knockdown the *f3a* gene by MO and investigate the effects on bleeding and angiogenesis.

## Methods

### Phylogenetic Analysis

TF protein and DNA sequences from human (*Homo sapiens*), rabbit (*Oryctolagus cuniculus*), mouse (*Mus musculus*), and zebrafish (*Danio rer*io) were retrieved from the National Center for Biotechnology Information (NCBI), European Molecular Biology Laboratory (EMBL), and Ensembl genome databases. Sequence alignment and construction of phylogenetic tree based on neighbor-joining method was performed using ClustalW2 (Hung and Weng, 2016).

### Zebrafish Strains and Maintenance

The following zebrafish lines were used in this study: Tübingen (ZIRC [Zebrafish International Resource Center], Eugene, Oregon), *Tg* (*fli1a:nEGFP*)*y7* (*Tg*(*gata1:dsRed*)*sd2* (Eisa-Beygi et al., 2018), *and Tg*(*kdrl:mCherry-CAAX*)*y171*(Borovina et al., 2010). Adult fish were maintained under a constant temperature of 28.0 °C and were subjected to 14-h light: 10-h ark photoperiod. All animal studies performed in these facilities were under the Medical College of Wisconsin–approved protocol for Animal Use Application. Freshly fertilized embryos were procured through natural breeding of adult zebrafish and were kept at 28.0°C in 1X E3 embryo medium (E3 medium) containing 5 mmol/L NaCl, 0.17 mmol/L KCl, 0.33 mmol/L CaCl_2_, 0.33 mmol/L MgSO_4_, and 0.05% methylene blue. In some instances, embryos were treated with 0.003% of 1-phenyl-2-thiourea (Sigma-Aldrich), starting at 24 h postfertilization (hpf), to minimize pigmentation.

### Embryo Collection and RNA Extraction

Wild-type strain zebrafish were maintained at 23.5°C on a 14 h light/10 h dark cycle. At the time of mating, breeding males and females were separated overnight before letting them spawn naturally in the morning to allow for synchronization of developmental stages. Fertilized eggs were grown at 28.0°C and staged using previously defined criteria (Kimmel et al., 1995; Machikhin et al., 2020). Samples from 9 different developmental stages, 2 hpf to 72 hpf, were collected by snap freezing the embryos in dry ice.

RNA extraction was performed as described by De Jong et al. (2010). In brief, tubes with embryos from different timepoints were kept in liquid nitrogen and ground individually with a liquid nitrogen pre-chilled metal micro-pestle (Carl Roth). The pestle was lifted slightly and 200 μl Qiazol (Qiagen, United States) was added. The pestle was placed back into the tube with Qiazol and the homogenate was allowed to thaw. Before removal, the pestle was washed with an additional 100 μl Qiazol to rinse off any material that might have stuck to the pestle. The homogenate was vortexed vigorously for 30 s, left at room temperature for at least 5 min, and then spun down quickly for 30 s 60 μl chloroform was added to the homogenate, vortexed for 15 s and kept at room temperature for 3 min. The partly separated mixture was transferred to a pre-prepared phase-lock gel heavy containing tube and centrifuged for 15 min at 12,000 × g. The aqueous phase was transferred to a new 1.5 ml tube. RNA was purified by column precipitation according to the RNeasy MinElute Cleanup kit (Qiagen, United States). At the end of the procedure, RNA was eluted in 12 μl nuclease-free water.

### *f3a* Expression by RT-PCR

Double-stranded cDNA was synthesized using the QuantiTect Reverse transcription kit (Qiagen, United States). For RT-PCR analysis of *f3a*, the forward and reverse primers used were 5′- ACG​TGG​AGT​CCA​AAA​CCA​AC -3′ and 5′- CAG​CGC​TGT​AAT​AGG​CCT​TC -3′ and for Beta-actin (control), 5′- CGA​GCT​GTC​TTC​CCA​TCC​A -3′ and 5′- TCA​CCA​ACG​TAG​CTG​TCT​TTC​TG -3′. Transcript levels were quantified by SYBR™ Green PCR Master Mix (IDT, United States). For amplification by PCR, the initial denaturing step at 94°C for 5 min was followed by 40 amplification cycles of 30 s at 94°C; 30 s at 60°C; 60 s at 72°C, and a final extension period of 10 min at 72°C. *f3a* expression at different embryonic stages was calculated using beta-actin as a normalizing control and the ΔΔCt method.

### Morpholino Approach

An antisense MO targeting the exon-intron junction (exon 3) of *f3a* (MO:*f3a*) was designed to knockdown the expression of *f3a*. As a negative control, we used a standard control MO (control-MO) specific to a human β-globin intron mutation. All MO solutions were synthesized by GeneTools (Oregon, United States). The MO sequences are as follows:

*f3a*-MO: 5′- AAA​CAA​CTA​AAC​ACT​GAC​TGT​CAT​G-3′

Control-MO: 5′-CCT​CTT​ACC​TCA​GTT​ACA​ATT​TAT​A-3′

All MO solutions were briefly heated at 65°C and resuspended in 1X Danieau buffer (58 mmol/L NaCl, 0.7 mmol/L KCl, 0.4 mmol/L MgSO4, 0.6 mmol/L Ca(NO_3_)_2_, 5.0 mmol/L HEPES, pH 7.6), and 0.1% (w/v) phenol red dye (Sigma-Aldrich, United States), to a final concentration of 8 ng/nl. Embryos at 1 to 2-cell stage were positioned in individual grooves made on a 1.0% agarose gel and were initially injected at concentrations ranging from 0.5 to 2 ng/nl.

### Screening of *f3a* Knockdown by PCR and Quantification

Total RNA extraction (De Jong et al., 2010) and cDNA synthesis were performed from control- and f3a- MO injected embryos as described before (Peterson and Freeman, 2009). For PCR-based gene expression analysis of f3a, the forward and reverse primers used were 5′- ACG​TGG​AGT​CCA​AAA​CCA​AC -3′ and 5′- TCC​GTC​ACA​TGC​AGC​TTT​GT -3′; for f3b, 5′- CTT​GGG​GAC​CCA​AAC​CTG​TC -3′ and 5′- TCC​AGT​CGG​TTA​AAC​TCC​GC -3′, and for GAPDH (control), 5′- GTG​GAG​TCT​ACT​GGT​GTC​TTC -3′ and 5′- GTG​CAG​GAG​GCA​TTG​CTT​ACA -3′. Each amplification reaction was separated on a 1.5% agarose gel stained with ethidium bromide-stain and the bands were visualized using UV Transilluminator (Chemidoc System, United States). Gels were quantified using ImageJ software (Schneider et al., 2012).

### Hemoglobin Staining

As described by Paffett-Lugassy and Zon (Paffett-Lugassy and Zon, 2005), O-dianisidine staining was used to detect the presence of hemoglobin within intact 52 hpf zebrafish embryos injected with Control or *f3a* MOs. Dechorionated or hatched embryos were stained in the dark for 30 min at room temperature within a solution containing O-dianisidine (0.6 mg/ml), 0.01 M sodium acetate (pH 4.5), 0.65% H_2_O_2_, and 40% (vol/vol) ethanol. After staining, embryos were washed with RO water and then fixed in 4% paraformaldehyde (PFA) for 1 h at room temperature. Pigments were removed from fixed embryos by incubating in a solution of 0.8% KOH, 0.9% H_2_O_2_, and 0.1% Tween-20 for 30 min at room temperature. Embryos were then washed with phosphate-buffered saline (PBS) containing 0.1% Tween-20, and then fixed again in 4% PFA for 3 h before storage in PBS at 4°C. All embryos were positioned in dorsal recumbency and imaged using a Keyence BZ-X700 fluorescent microscope (Japan).

### Vascular Development

Endothelial-specific transgenic lines of zebrafish (*flk1:egfp-NLS/kdrl:mCherry-CAAX*) were used to study vascular development. In brief, *Tg* (*fli1a:nEGFP*)y7 (*Tg*(*gata1:dsRed*)*sd2* (Eisa-Beygi et al., 2018), *and Tg*(*kdrl:mCherry-CAAX*)*y171*(Borovina et al., 2010) lines were maintained at 23.5°C on a 14 h light/10 h dark cycle. At the time of mating, breeding males and females were separated overnight before letting them spawn naturally in the morning. Embryos at 1 to 2-cell stage were positioned in individual grooves made on a 1.0% agarose gel and were initially injected at concentrations ranging from 1 to 2 ng/nl.

### Microscopy

For bright-field/fluorescent microscopy, wild-type or transgenic embryos were first embedded in 1.0% low melting agarose in E3 medium and 30 μg/ml tricaine mesylate. Embryos were mounted on 35 mm glass-bottom Petri dishes and imaged using Keyence BZ-X700 fluorescent microscope (Japan). A Texas Red filter cube (OP-87765, Keyence) was used to detect mCherry and dsRed (red fluorescent protein)-labeled cells, a GFP filter cube (OP-87763, Keyence) was used to detect GFP/EGFP-labeled tissues, and a 4′,6-diamidino-2-phenylindole (DAPI) filter cube (OP-87762, Keyence) was used to image DAPI-stained samples. Z-series bright-field and fluorescent images were acquired, and composite images were generated using the BZ-X Image Analyzer software. Brightness and contrast were adjusted using Fiji Software.

### Statistical Analysis

Statistical analysis was performed using GraphPad Prism 8 software (GraphPad Software, United States) by Student’s t test (2 groups; nonparametric). Bleeding and morphological phenotypes were expressed as percentage differences from respective controls.

## Results

### Phylogenic Analysis and Protein Identity of Zebrafish TF-*f3b*

**FIGURE 1:**
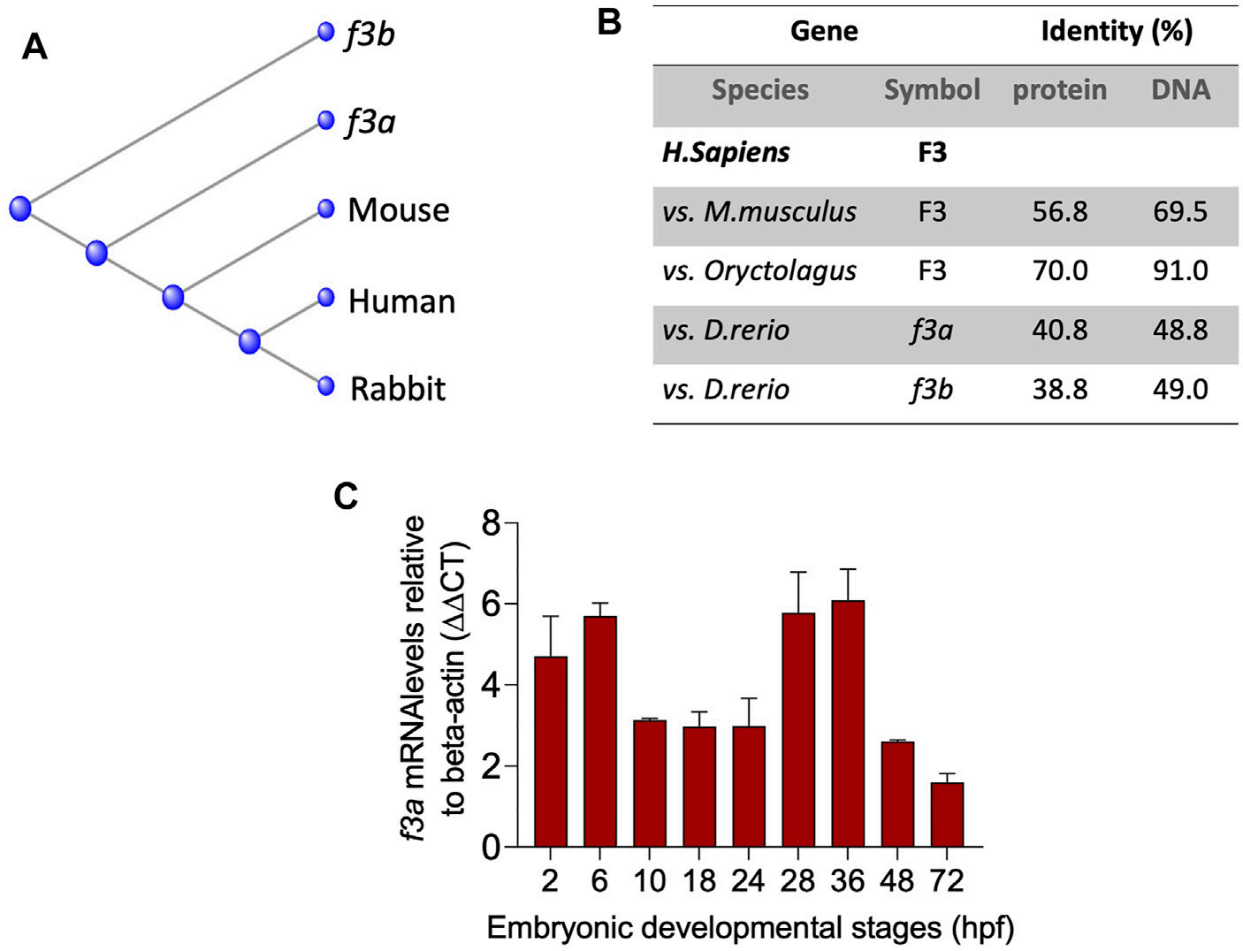
Evolutionary relationships and Spatiotemporal expression of Zebrafish TF-*f3b:* **(A)** Phylogenetic tree of tissue factor (F3) from zebrafish (Danio), mouse (Mus), rabbit (Oryc), and human (Homo). **(B)** Protein and DNA percent identity of F3 from zebrafish (Danio), mouse (Mus), rabbit (Oryc), and human (Homo). **(C)** mRNA expression from whole embryos demonstrates that zebrafish *f3a* is expressed in early embryonic stages. Beta-actin was used to normalize the expression of *f3a*.

To understand the evolutionary relationships across different species, we compared the mouse, human and rabbit TF gene and protein sequences to the TF paralogs (*f3a* and *f3b*). Multiple sequence alignment (ClustalW) revealed that zebrafish *f3a* protein was more closely related to that of mouse and human compared with *f3b* (Figure 1A) and was more identical to human F3 compared to *f3b* (Figure 1B). RT-PCR analysis of expression during embryonic developmental stages showed that *f3a* was detected as early as 2 h (64-cell stage) and in larvae at up to 36 hpf (Figure 1C). Previous report indicate detecting *f3a* transcripts at 64-cell stage, which suggest maternal contribution to larvae at this stage (Kimmel et al., 1995). Between 2.75 hpf to 6 hpf, both maternal and zygotic expression appears to be present because *f3a* was strongly transcribed compared to earlier (2 hpf) and later stages (10, 18, and 24 hpf) (Figure 1C). These observations indicate *f3a* transcription crossover from maternal to zygotic stages (between 2 hpf to 6 hpf). From 28 hpf to 36 hpf the expression of *f3a* was relatively stable, suggesting that *f3a* expression contributes to the development of yolk sac, circulation, pigmentation, heartbeat, circulation in the aortic arch, and fins (Kimmel et al., 1995). At 48 hpf and 72 hpf expression was low compared to 36 hpf, suggesting *f3a* might not play a significant role in hatching and protruding mouth stages.

### Spatiotemporal Expression of Zebrafish TF-*f3a*

**FIGURE 2:**
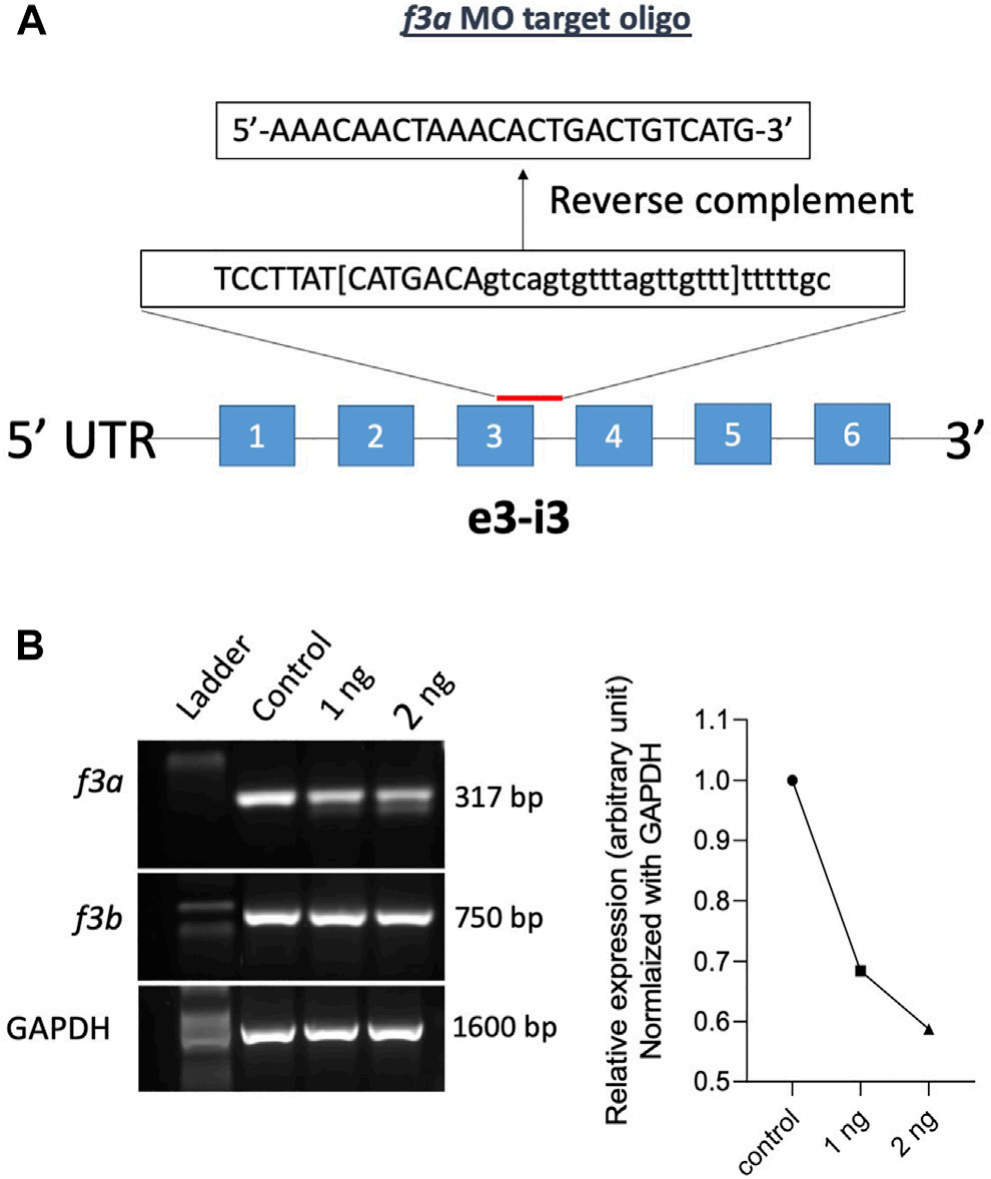
*f3a* knockdown by Morpholino approach: **(A)** schematic diagram of *f3a* antisense morpholino oligonucleotide (MO) targeting the exon-intron junction (exon 3); design and synthesis by GeneTools (Oregon, United States). **(B)** The molecular targeting and efficiency of *f3a* MO-injected with 1 and 2 ng of *f3a* was assessed by PCR and quantified using ImageJ.

Previously, Zhou and colleagues reported that *f3b* knockdown in zebrafish exhibits defective vasculogenesis (Zhou et al., 2011). Because f3a′s role during embryonic development was not known, we therefore examined its role by mRNA knockdown using MO antisense oligonucleotides (Gene Tools, United States) (Figure 2A). Embryos were collected freshly and 1 and 2 ng of *f3a* MO, targeted exon-intron junction (*e3-i3*), was injected at 1- to 2-cell stage. Twenty-four hours later, RNA was isolated from the injected embryos and expression of *f3a* and *f3b* analyzed by PCR. Control group embryos were injected with a random sequence of standard MO. Our data revealed lower PCR transcript levels of *f3a*, but not *f3b*, at both 1 and 2 ng levels in injected embryos, suggesting specificity of the targeted knockdown towards *f3a* (Figure 2B).

### *f3a* Knockdown by Morpholinos Approach and Morphological Characterization

**FIGURE 3:**
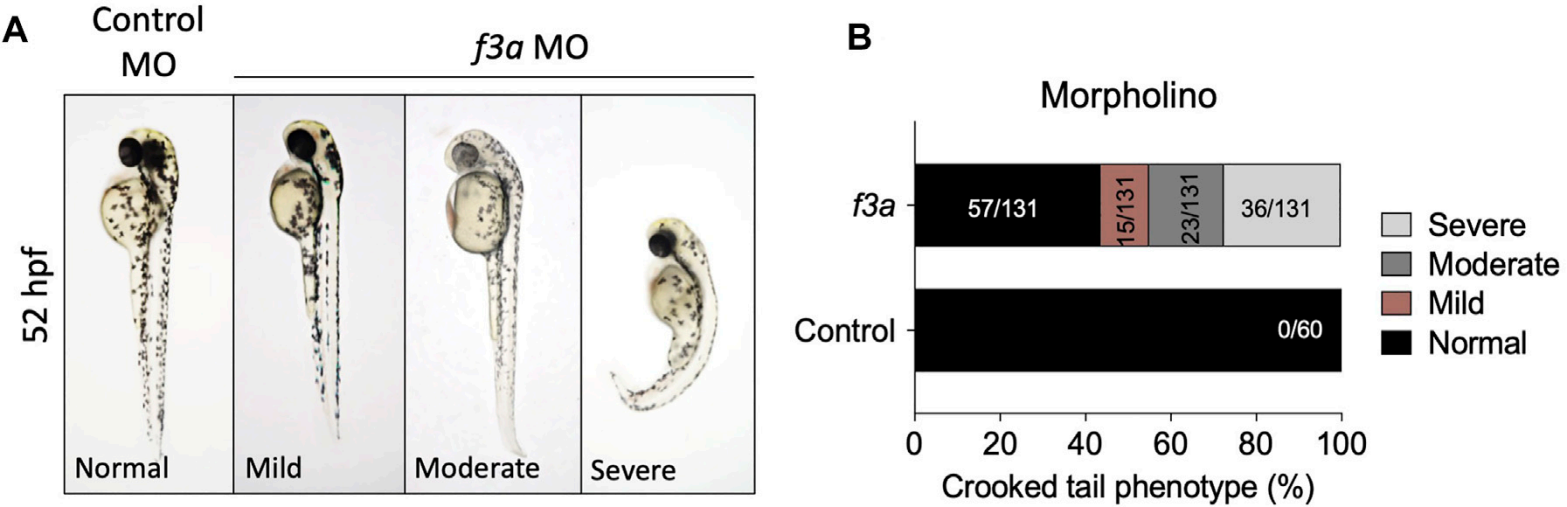
MO-mediated knockdown of *f3a* expression results in morphological abnormalities: **(A)** Zebrafish embryos at 1-or 2-cell stage were injected with control and *f3a* MO and examined morphologically at 52 hpf. MO phenotypes: control MO injected (control MO), and mild, moderate, and severe phenotypes. **(B)** The morphological abnormalities were quantified. Numbers in the bars represent the ratios used to calculate the percentages. Data were pooled from three independent experiments.

The *f3a* morphants, injected with 2 ng of MO, were examined at 52 hpf for any defect in morphological appearance. Control MO-injected embryos appeared to be normal, with properly developed head and eyes, and elongated body axis, and a long tail (Figure 3A). In contrast, embryos injected with 2 ng of *f3a* MO exhibited a shortened body axis and a short, crooked tail. Based on the degree of severity, we segregated them as mild, moderate, and severe and quantified the severity. On an average, 11.45% (15/131) showed mild phenotype with shortened body axis. Moderate defect in morphological appearance was observed in 17.55% (23/131), with a partially curved tail. Severe morphological defect was observed in 27.5% (36/131) of embryos, with curved body axis and defectively developed tail (Figure 3B).

### Bleeding Phenotype in *f3a* Knockdown Zebrafish

**FIGURE 4:**
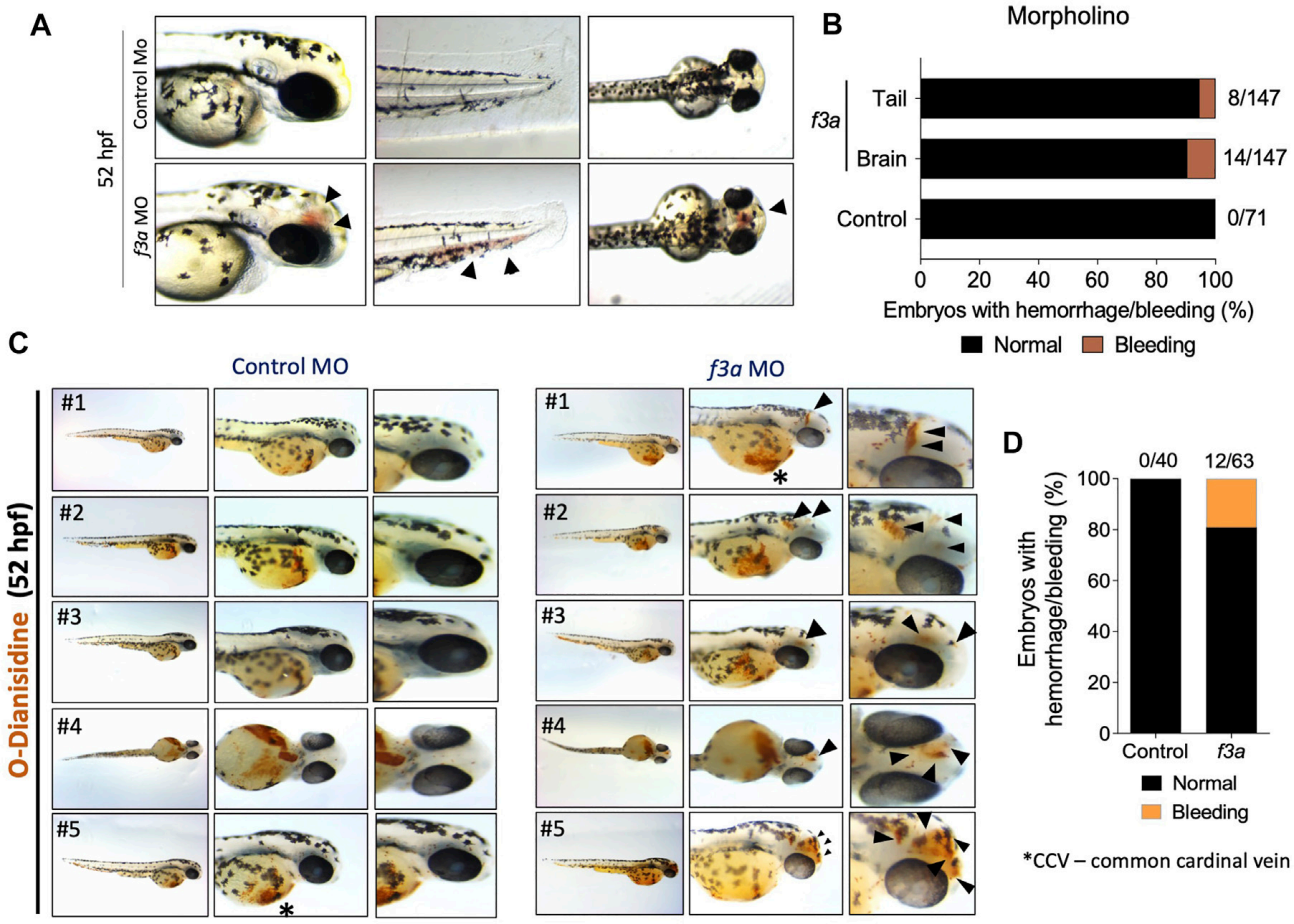
*f3a* knockdown shows bleeding phenotype: **(A)** Lateral and dorsal views of head, and lateral view of tail in live embryos. The arrows depict brain and tail bleeding at 52 hpf and **(B)** quantification of the same. Numbers in the bars represent the ratios used to calculate the percentages. **(C)** Bright-field images of zebrafish embryos stained with O-dianisidine (OD) and imaged at 48 hpf. Black arrows indicate sites where abnormal accumulation of hemoglobinized blood was detected in the head region (control MO and *f3a* MO). **(D)** Percentage of cerebral hemorrhage in embryos at 52 hpf. Numbers in the bars represent the ratios used to calculate the percentages. Data were pooled from three independent experiments.

As TF is a triggering factor for activation and coagulation cascade, in a separate experiment, we checked whether the knockdown of *f3a* showed bleeding phenotype. We observed embryos at 52 hpf, which showed bleeding mostly in the head and tail regions (Figure 4A). Control MO-injected embryos appeared normal with no visible bleeding. Conversely, *f3a* MO-injected embryos showed 5.44% (8/147) and 9.52% (14/147) bleeding in tail and head regions, respectively (Figure 4B). With the next set of *f3a* and standard MO injection, at 52 hpf, we stained the embryos with O-dianisidine to detect hemoglobin-containing cells (Figure 4C); around 7.5% had bleeding in the head. No bleeding was observed in control MO-injected embryos at 52 hpf (Figures 4C,D). Common cardinal vein (CCV) region also stained with O-dianisidine due to the presence of a pool of blood cells and heart. This region appears stained irrespective of phenotype, and therefore, we did not consider the CCV region for bleeding score. These findings confirm that, similar to *f3b* (Zhou et al., 2011), *f3a* plays a role in vascular stability in our zebrafish model.

### Defective Angiogenesis in *f3a* Knockdown Zebrafish

**FIGURE 5:**
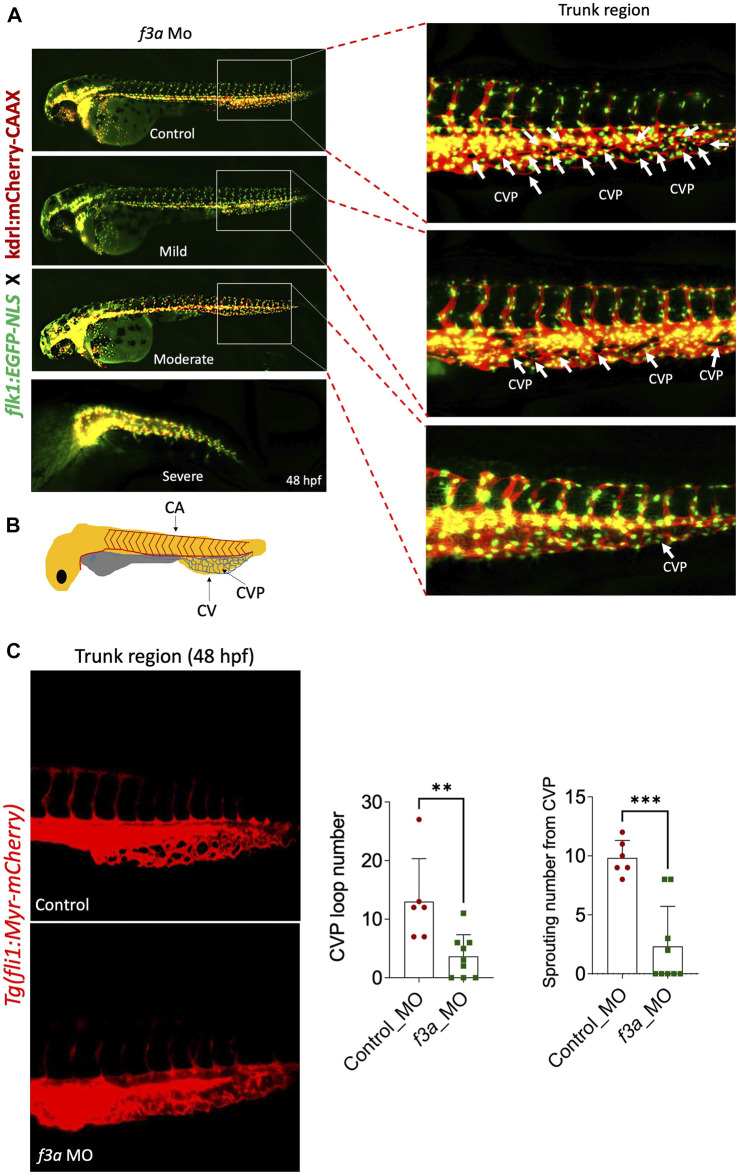
*f3a* knockdown shows delayed angiogenesis: To study vascular development, endothelial-specific transgenic lines of zebrafish (*flk1:egfp-NLS/kdrl:mCherry-CAAX*) embryo were used to knockdown *f3a*. **(A)** Images represent MO-mediated knockdown of *f3a* expression resulting in morphological abnormalities. MO phenotypes: control MO injected (control MO), and mild, moderate, and severe phenotypes. Differences in caudal vein plexus (CVP) in the trunk regions are indicated by white arrows. **(B)** Schematic diagram depicts to identify Caudal vein (CV), caudal vein plexus (CVP) and Central aorta (CA). **(C)** *Tg(fli1:Myr-mCherry)* line was used to quantify CVP loop number and Sprouting number from the CVP at 48 hpf. Embryos were randomly picked from ∼60 MO-injected (6 from control and 9 from *f3a* MO) embryos from control and *f3a*.

Next, we investigated the effect of *f3a* knockdown on vasculogenesis and angiogenesis using endothelial-specific transgenic lines of zebrafish (*flk1:egfp-NLS/kdrl:mCherry-CAAX*) and imaged vascular development. Interestingly, we did not observe any defect in vessel formation at 48 hpf between control and *f3a*-MO-injected groups (Figure 5A), suggesting that *f3a* might not be involved in initial development of central aorta (CA) and no major deficit in vasculogenesis. During zebrafish embryonic angiogenesis, caudal vein plexus (CVP) and inter-segmental vessel (ISV) formation are the two obvious vessel patterns observed. In *f3a* knockdown embryos, the development of CVP was greatly abrogated (Figure 5C). Quantification of loop formation at mean CVP showed a 3-fold decrease in *f3a* morphants versus controls at 48 hpf (Figure 5C). The CVP forms during a very active period of angiogenic sprouting from beginning at 25 hpf when venous endothelial cells of the posterior cardinal vein sprout and migrate ventrally, then fuse with neighboring tip cells (Figure 5B). Thus, we looked at the difference in the CVP angiogenic sprouting at 48 hpf. Like number of CVP, the mean spouting from CVP was 4-fold lower in *f3a* injected MO than the control MO-injected group (Figure 5C). Occasionally, we also observed a defect in central arteries (CtAs) and primordial midbrain channel (PMBC) in *f3a* morphant CVP defective embryos. However, we did not see any impairment in the intersegmental vessel (ISV) development. These findings support the notion that *f3a is* required for angiogenesis.

## Discussion

In the present study, we used a zebrafish knockdown model to study one of the homologs genes of TF (*f3a*) to gain insights into the developmental aspects. Previously, Zhou and his colleagues investigated the role of *f3b* in zebrafish by morpholino approach and reported that *f3b* knockdown in zebrafish exhibits defective vasculogenesis (Zhou et al., 2011). In this study, we successfully knocked down *f3a* by morpholino approach and investigated its effects on bleeding and vascular development.

Phylogenetic analysis revealed that *f3a* is more closely related to humans and mice than *f3b*. Spatiotemporal expression of *f3a* by RT-PCR revealed that unlike *f3b* (expressed only after 18 hpf), *f3a* expression occurs across all the stages, suggesting that *f3a* transcripts are both maternally and zygotically expressed. Expression of *f3a* is stable throughout the embryonic stages. *f3a* MO-injected embryos showed morphological abnormalities, including shorter body lengths and bent tails. The standard MO-injected embryos had no phenotype changes.

Evaluating the vascular development using endothelial-specific transgenic lines of zebrafish (*flk1:egfp-NLS/kdrl:mCherry-CAAX*) revealed early vascular development (central aorta and ISV) remain stable even after *f3a* knockdown. Interestingly, the development of CVP, which is composed of a dorsal and ventral vein with interconnecting vessels, and spouting from CVP were suppressed in the zebrafish *f3a* knockdown models, signifying the contribution of *f3a* in angiogenesis. Despite defective angiogenesis, knockdown of *f3a* also resulted in mild bleeding in the brain and tail regions. Similar bleeding characteristics were observed in zebrafish *f3b* knockdown models (Zhou et al., 2011).

In conclusion, similar to *f3b* knockdown (Zhou et al., 2011), *f3a* knockdown also showed a mild bleeding phenotype, suggesting that both *f3a* and *f3b* are required for effective hemostasis function in zebrafish. Our study also confirmed that *f3a* is essential for angiogenesis in addition to its involvement in hemostasis. In addition, although we see a stable formation of central aorta, which implies no defect in vasculogenesis, at 48 hpf in control and *f3a*-MO-injected groups, it must be further validated at earlier time points (12–18 hpf). Nevertheless, a rescue phenotype validation with zebrafish *f3a* or human F3 or a CRISPR/cas9-mediated gene knockout approach would provide more insights to understand the hemostasis and non-hemostasis functions of TF paralogs (*f3a* and *f3b*) in zebrafish. Further studies are required to validate the *f3a-*knockdown-mediated bleeding phenotype is blood-vessel autonomous or resulting from abnormal coagulation.

## Data Availability

The raw data supporting the conclusion of this article will be made available by the authors, without undue reservation.
